# Pediatric blood cultures—turning up the volume: a before and after intervention study

**DOI:** 10.1007/s00431-024-05544-0

**Published:** 2024-04-24

**Authors:** Seán Olann Whelan, Conor Mulrooney, Frank Moriarty, Martin Cormican

**Affiliations:** 1 National Virus Reference Laboratory, University College Dublin, Dublin, Ireland; 2 Division of Medical Microbiology, Galway University Hospital, Galway, Ireland; 3 Department of Microbiology, Children’s Health Ireland at Temple Street, Dublin, Ireland; 4 School of Pharmacy and Biomolecular Sciences, RCSI University of Medicine and Health Sciences, Dublin, Ireland; 5 Discipline of Bacteriology, College of Medicine, Nursing & Health Sciences, University of Galway, Galway, Ireland

**Keywords:** Bacteremia, Blood cultures, Diagnostic accuracy, Quality improvement

## Abstract

The major determinant of blood culture (BC) diagnostic performance is blood volume, and pediatric sample volumes are frequently low. We aimed to assess BC volumes in our institution, design an intervention to increase volumes, and assess its impact. All pediatric BCs submitted over a 7-month period to the microbiology laboratory in a university hospital (including emergency department, pediatric ward, and neonatal unit) were included. A pre-intervention period assessed current practice. A multi-faceted intervention (education, guideline introduction, active feedback strategies) was collaboratively designed by all stakeholders. Impact was assessed in a post-intervention period. The main outcome measures included the percentage of samples adequately filled using three measures of sample adequacy (1) manufacturer-recommended minimum validated volume—> 0.5 ml, (2) manufacturer-recommended optimal minimum volume—> 1.0 ml, (3) newly introduced age-specific recommendations. Three hundred ninety-eight pre-intervention and 388 post-intervention samples were included. Initial volumes were low but increased significantly post-intervention (median 0.77 ml vs. 1.52 ml), with multivariable regression analysis estimating volumes increased 89% post-intervention. There were significant increases in all measures of volume adequacy, including an increase in age-appropriate filling (20.4–53.1%), with less improvement in those aged > 3 years. Overall, 68.4% of pathogens were from adequately filled cultures, while 76% of contaminants were from inadequately filled cultures. A pathogen was detected in a higher proportion of adequately filled than inadequately filled cultures (9.4% vs. 2.2%, *p* < 0.001).

*  Conclusion*: Blood volume impacts BC sensitivity, with lower volumes yielding fewer pathogens and more contaminants. Focused intervention can significantly improve volumes to improve diagnostic performance.

**Table:** 

**What is Known:** *• Blood volume is the major determinant of blood culture positivity, and yet pediatric blood culture volumes are frequently low, resulting in missed pathogens and increased contamination.*	
**What is New:** *• Adequately filled (for age) blood cultures have a pathogen detection rate three times higher than inadequately filled blood cultures.* *• This interventional study shows that collaboratively designed multi-modal interventions including focus on accurate volume measurement can lead to significant increases in blood volumes and improve blood culture diagnostic performance.*

**Supplementary Information:**

The online version contains supplementary material available at 10.1007/s00431-024-05544-0.

## Introduction

Bloodstream infections (BSI) are associated with significant morbidity and mortality in children [1, 2]. Blood cultures (BCs), incubated using automated systems, remain the gold standard for diagnosing BSI [3–5].

Culturing pathogens has many benefits. It confirms a diagnosis, suggests potential infection sources, permits susceptibility testing, and in some cases, directing additional investigations or treatment durations [6, 7]. There are also system benefits, including surveillance of antimicrobial resistance, central-line care, and assessing empiric antimicrobial guidance appropriateness [8, 9]. Negative cultures are also significant, and are frequently used, alongside clinical assessment, to stop antimicrobials [10].

There are several factors that affect BC performance, including time from venipuncture to incubation [11, 12], relative fastidiousness of the organism [13], and the timing and quantity of BC taken [10, 14, 15]. The major determinant of BC positivity however is the blood volume inoculated into the BC, with higher inoculums correlating with increased pathogen detection [4, 15–20]. The detection of bacteremia by modern culture systems is dependent on the bottle blood-to-broth ratio, with optimal ratios targeted to improve test performance [4].

Given difficulties in obtaining large blood volumes in children, pediatric bottles, validated for volumes as low as 0.5 ml, have been introduced [13]. These may improve rates and timeliness of pathogen detection at lower inoculum volumes. Some studies have suggested these manufacturer-recommended “minimum” blood volumes may lead to under-detection of bacteremia, particularly at low bacterial concentrations, which are common and clinically relevant in pediatric sepsis [16, 17]. Failing to detect pathogens in the presence of clinical signs of sepsis often leads to the label of culture-negative sepsis. Interestingly, in neonates, culture-negative bacteremia cases had lower bacterial loads than culture-positive cases [21], suggesting, in at least some of these cases, pathogens may not be detected due to insufficient sample volumes at low bacterial concentrations.

While in adults there are accepted blood volume recommendations, this is not the case in pediatrics. There are varied recommendations based on heterogenous age or weight-based categories, or percentage blood volumes, with at least 12 distinct approaches (Online Supplemental Table 1) [4, 13, 14, 16, 18, 22–28]. There are several problems with this variation. There are no comparisons between schemas, and many studies have derived their own, limiting comparability. Some recommendations recommend blood volumes above that safely replaced by normal hematopoiesis [29]. Finally, some high target blood volumes are potentially not practical in pediatrics, due to the often-challenging nature of venipuncture in children, and the need to send other tests in a single blood draw.

Given the primacy of sample volume in BC performance, the first aim of this study was to quantify the blood volumes of BCs in our institution. Secondly, we sought to design and implement a quality improvement intervention to improve volumes. Finally, we monitored the impact of this intervention post-implementation.

## Methods

### Study setting

Galway University Hospital (GUH) is a teaching hospital in the west of Ireland, incorporating an emergency department (ED), pediatric ward, and a neonatal unit (NNU). During the study period, only BD BACTEC^™^ Peds Plus^™^ (Becton Dickinson & Company, New Jersey, USA) BCs were in use in all areas [13]. At the study outset, there was no specific recommendations on blood volumes other than manufacturer recommendations. No formal sample size calculation was conducted. Study timelines were designed to ensure completion during one non-consultant hospital doctor rotational training year, to minimize changes in sample takers between pre- and post-implementation phases. Ethical approval for the study was received from the GUH Audit Committee.

### Sample processing

On receipt from the supplier, each BC was identified by barcode and weighed using calibrated *Sartorius BP310P* (Sartorius, Göttingen, Germany) balance, before delivery to clinical areas. Inoculated BCs were incubated in the BACTEC^™^ FX BC System (Becton Dickinson & Company, New Jersey, USA) and weighed within 24 h of receipt. Unfilled bottle weight was subtracted from the filled bottle weight, and cap weight of removable cap (0.41 g) was added. This was converted to volume using the weight-to-volume conversion factor for blood (1.0506 g/ml) [30]. The number of gradation marks filled on the vial label was also noted.

All BCs received in the study period were included. Request forms were reviewed to record sample taker grade (Nurses, Senior House Officer [SHO], or Registrar), clinical area, sampling site (line or peripheral) and patient age at sampling. Patient weight, blood culture results, and for NNU patients, corrected gestational age at sampling were obtained from electronic records. Isolates were considered either pathogens or contaminants based on the contemporaneous consensus assessment of pediatric and microbiology teams based on clinical judgement of each individual case.

### Pre-intervention review and intervention design

A pre-intervention phase to establish practice was performed between October 2022 and January 2023, with sample takers unaware of sample weighing. Following this, interim analysis was performed and shared with sample takers. A formal guideline, based on the schema proposed by both Connell et al. and Harewood et al. (Online Supplemental Image 1), was designed collaboratively by all stakeholders [26, 31]. This was selected as it was the only schema used in more than a single study and was considered to balance practical targets, while significantly increasing blood volumes obtained. Guidance was displayed prominently in all areas where BCs are stored and taken in clinical areas. An education program, focusing on BC volumes and the newly introduced guidance, for all stakeholders across all three clinical areas was undertaken. A review of BC-sampling processes was also undertaken, and some changes were made (e.g., exchanging 1 ml for 3 ml syringes in BC sets).

### Post-interventional phase

During this phase (January–May 2023), regular guidance reminders were provided via attendance at department meetings, safety pauses, and NNU stewardship rounds, as well as through a text-based communication tool already in use. Sample takers were also asked to indicate on the request form estimated blood volume obtained. Regular volume feedback was given to sample takers. This included at the individual sample taker level, where estimated and real volumes taken were provided to sample takers on specific samples, and at the group level, where performance over the preceding week was presented to clinical areas at these guidance reminders. Feedback allowed us to flag individual sample users for further support and training, and to raise ongoing issues at teaching.

### Analysis and statistics

Characteristics of patients and BCs were summarized overall, and during pre- and post-intervention periods. Wilcoxon rank-sum and chi-squared tests were used to assess whether characteristics differed between periods (for non-normally distributed continuous and categorical variables, respectively). Blood volumes were summarized by characteristics of patients and BCs and associations between characteristics and blood volume were tested. Pearson correlation coefficients (PCC) were determined between blood volumes and gestational age and weight. Rate of contaminants and significant results were examined across levels of filling. Blood volumes were summarized during both periods using median and inter-quartile range, and blood volume and proportions of cases adequately filled were compared between the pre- and post-intervention periods using Wilcoxon rank-sum and chi-squared tests. Three measures of volume adequacy were summarized and compared between phases: (1) ≥ 0.5 ml (minimum as per manufacturer guidance), (2) ≥ 1 ml (optimal as per manufacturer guidance), and (3) newly introduced age-appropriate guidelines:

- < 1 month: ≥ 0.5 ml
- 1 month–3 years: ≥ 1 ml
- > 3 years: ≥ 4 ml

Multivariable regression analyses were used to estimate the effect of the intervention, controlling for characteristics which may have changed between study periods and related to blood volumes. Poisson with robust variance estimator and logistic regression models were used for the outcomes blood volumes and adequate filling, respectively. Lastly, to assess sensitivity of findings to any secular trend in BC filling, an interrupted time-series analysis was conducted, estimating the trend pre-intervention, any immediate change in the level of blood volume at intervention implementation, and any change-in-trend over the post-intervention period. All analyses were conducted using Stata with statistical significance defined as *p* < 0.05.

## Results

### Descriptive characteristics

Overall, 786 BCs (398 pre-intervention, 388 post-intervention) were included. The characteristics of included patients are shown in Table 1. The median age was 2.4 years, with a higher proportion of children > 3 years of age in the post-intervention phase (188/388, 48.5% vs. 167/398, 42.0%, *p* = 0.01). Most samples came from ED (464/786, 56.0%), with the remainder from the pediatric ward (213/786, 27.1%) and NNU (109/786, 13.9%).

**Table 1:** Descriptive characteristics of included blood cultures overall and for pre- and post-intervention periods

Characteristics	*N* (%, unless otherwise specified)			*p* value*
	Pre-intervention	Post-intervention	Total	
Number of blood cultures	398	388	786	
Number of patients	273	268	541	
Age (years), median (IQR)	2.0 (0.1–5.0)	2.7 (0.3–6.4)	2.4 (0.2–5.8)	0.24
**Age category**				0.12
< 1 month	74 (18.6%)	72 (18.6%)	146 (18.6%)	
1 month–3 years	157 (39.4%)	128 (33.0%)	285 (36.3%)	
> 3 years	167 (42.0%)	188 (48.5%)	355 (45.2%)	
**Location**				0.01
ED	248 (62.3%)	216 (55.7%)	464 (59.0%)	
NNU	62 (15.6%)	47 (12.1%)	109 (13.9%)	
Ward	89 (22.1%)	125 (32.2%)	213 (27.1%)	
Gestational age (weeks), median (IQR)	34.4 (32.9–39.3)	38.1 (35.1–40.0)	37.1 (33.3–39.9)	0.01
**Gestational age category**				0.20
Very preterm (< 32 weeks)	8 (12.7%)	3 (6.3%)	11 (9.9%)	
Moderate-late preterm (32–37 weeks)	27 (42.9%)	16 (33.1%)	43 (38.7%)	
Term (> 37 weeks)	28 (44.4%)	29 (60.4%)	57 (51.4%)	
**Weight** (kg)—overall, median(IQR)	12.1 (4.7–18.6)	14.0 (5.9–22.4)	13.4 (4.9–21.2)	0.01
**Weight** (kg)—NNU, median(IQR)	2.3 (1.6–3.3)	3.0 (2.0–3.6)	2.7 (1.8–3.4)	0.02
**Sampler**				0.27
Nurse	42 (10.7%)	37 (9.8%)	79 (10.2%)	
Registrar	201 (51.0%)	175 (46.2%)	376 (48.6%)	
SHO	151 (38.3%)	167 (44.1%)	318 (41.1%)	
**Site**				0.72
Peripheral	350 (87.9%)	338 (87.1%)	688 (87.5%)	
Central	48 (12.1%)	50 (12.9%)	98 (12.5%)	
**BC results**				
**Positive**	36 (9.0%)	27 (7.0%)	63 (8.0%)	
**Significance**				0.09
Contaminant	11 (30.6%)	14 (51.9%)	25 (39.7%)	
Pathogen	25 (69.4%)	13 (48.1%)	38 (60.3%)	

*p value for difference in patient characteristics between pre-intervention and post-intervention period, based on Wilcoxon rank-sum test and chi-squared test

Overall blood volumes per patient characteristics are summarized in Online Supplemental Table 2. As expected, younger children had lower volumes submitted, and accordingly, median volumes in NNU were lower than in other areas. In NNU, volume correlated poorly with both gestational age (PCC 0.11, *p* = 0.24) and weight-at-sampling (0.16, *p* = 0.08). Of infants, 90.1% (100/111) had a corrected gestational age of ≥ 32 weeks at the time of sampling, with twice as many very preterm infants in the pre-intervention phase.

BCs from central lines had higher volumes than peripheral cultures (median 2.10 ml [IQR 1.33–2.85 ml] vs. 1.05 ml [0.57–1.80 ml], *p* < 0.01). Most (688/786, 87.5%) BCs were peripheral. Most sample takers were doctors, with 10.2% (79/773) of samples taken by nursing staff. Nursing staff had higher fill volumes than doctors, likely reflecting sampling practices, with 65.8% (52/79) of nursing cultures coming from lines, and most from older children.

**Fig. 1:**
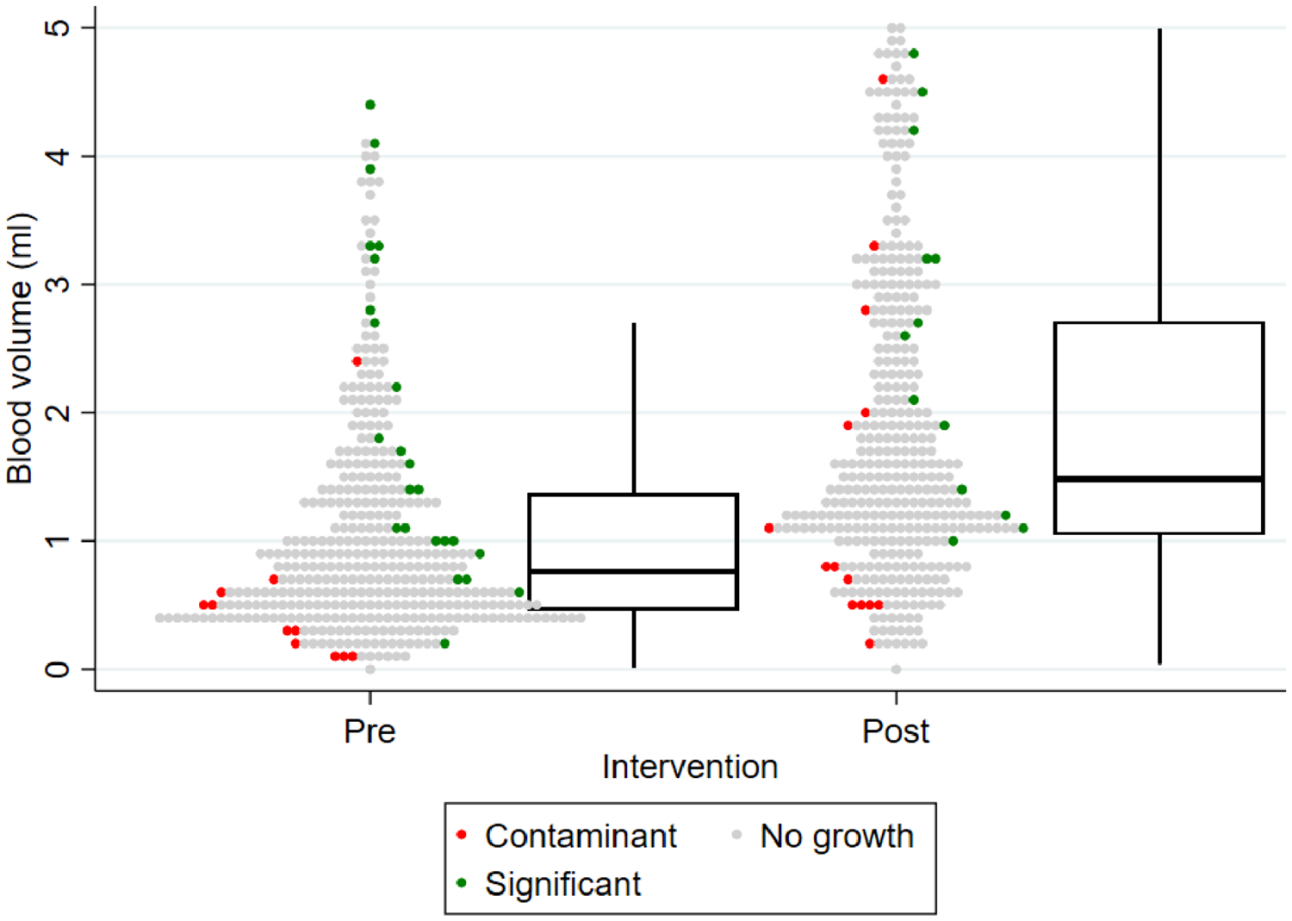
Distribution of blood culture volumes by result, divided into pre- and post-intervention phases. Each dot represents a single sample, colored according to blood culture result, as indicated by legend

The overall distribution of blood volumes by intervention phase and result are depicted in Fig. 1. There were 63 positive BCs, including a true-positive rate of 4.8% (38/786) and a contamination rate of 3.1% (25/786). Organisms isolated are outlined in Online Supplemental Table 3. There were no significant differences between positive and negative BC volumes. However, amongst positives, pathogen-isolating BCs had significantly higher volumes than contaminant-isolating BCs (1.85 ml [1.06–3.23 ml] vs. 0.54 ml [0.35–1.12 ml], *p* < 0.001). Overall, 68.4% (26/38) of true-positives were from adequately filled cultures, while 76% (19/25) of contaminants were from inadequately filled cultures. A pathogen was detected in 9.4% (27/287) of adequately filled BCs, compared to 2.2% (11/499) of inadequately filled cultures, while there was no significant difference amongst contaminant BCs (3.8%, 11/287 vs. 2.8% 14/499).

### Effect of intervention on blood volumes

As seen in Table 2, the pre-intervention median blood volume was 0.77 ml (IQR_0.46–1.39 ml), with 29.6% (118/398) < 0.5 ml, and 63.6% (251/398) < 1 ml. Only 20.4% (81/398) were adequately filled for age. Post-intervention, all measures improved significantly with a median volume of 1.52 ml (1.06–2.83 ml), 6.2% (24/388) < 0.5 ml, 22.7% (88/388) < 1 ml, and 53.1% (182/388) adequately filled for age (all *p* < 0.001).

**Table 2:** Blood volumes overall and for pre- and post-implementation periods

	Pre-intervention *n* = 398	Post-intervention *n* = 388	Total *n* = 786	*p* value
Blood volume (ml), median (IQR)	0.77 (0.46–1.39)	1.52 (1.06–2.83)	1.14 (0.60–2.01)	< 0.001
Adequacy measures, *n* (%)				
0.5 ml+	280 (70.4%)	364 (93.8%)	644 (81.9%)	< 0.001
1 ml+	147 (36.4%)	300 (77.3%)	445 (56.6%)	< 0.001
Age-specific guidance				
< 1 month	34 (45.9%)	69 (95.8%)	103 (70.5%)	< 0.001
1 month–3 years	41 (26.1%)	94 (73.4%)	135 (47.4%)	< 0.001
> 3 years	6 (3.6%)	43 (22.9%)	49 (13.8%)	< 0.001
Overall	81 (20.4%)	206 (53.1%)	287 (36.5%)	< 0.001

**Fig. 2:**
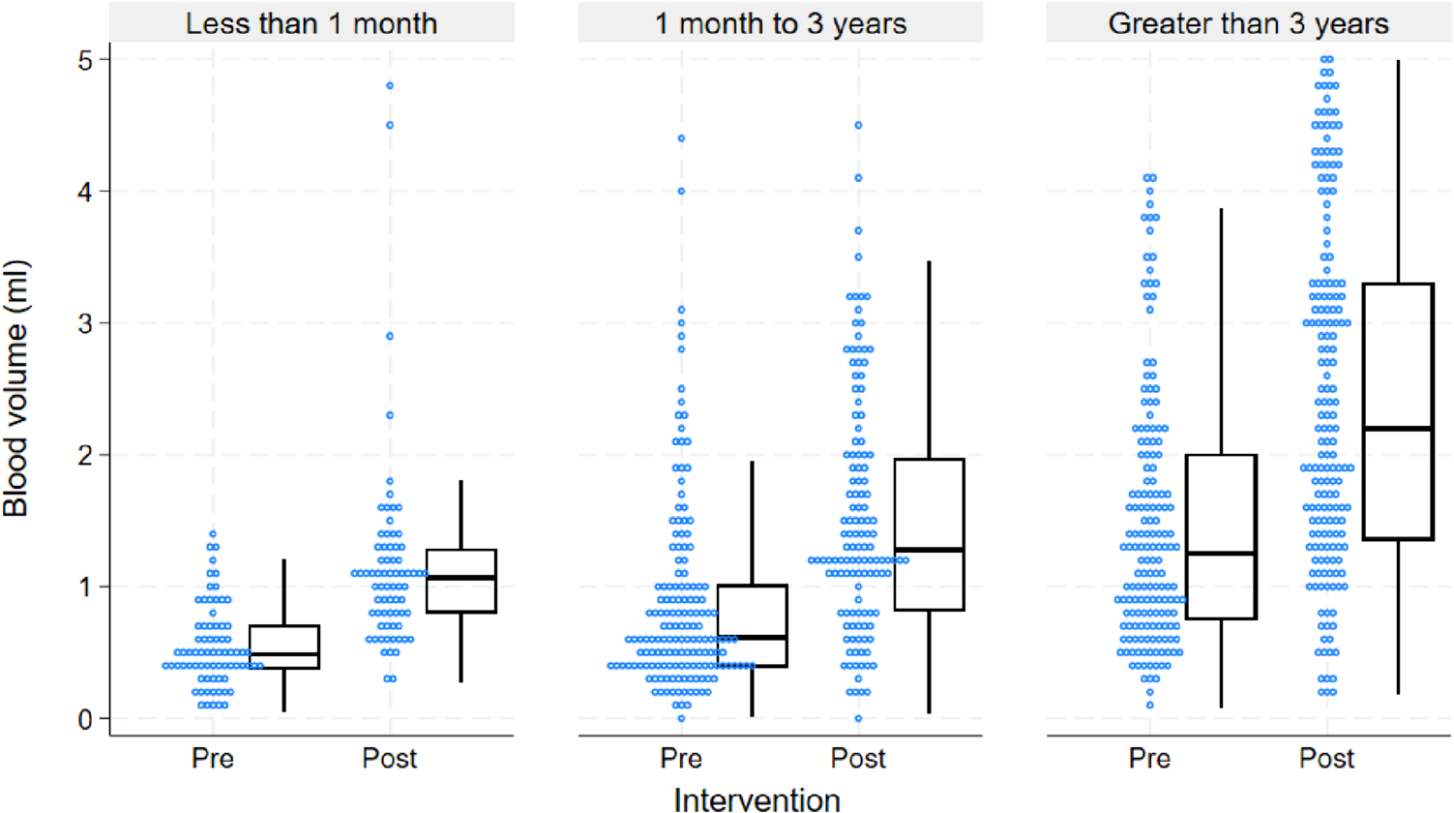
Blood culture blood volumes per age category, divided into pre- and post-intervention

**Fig. 3:**
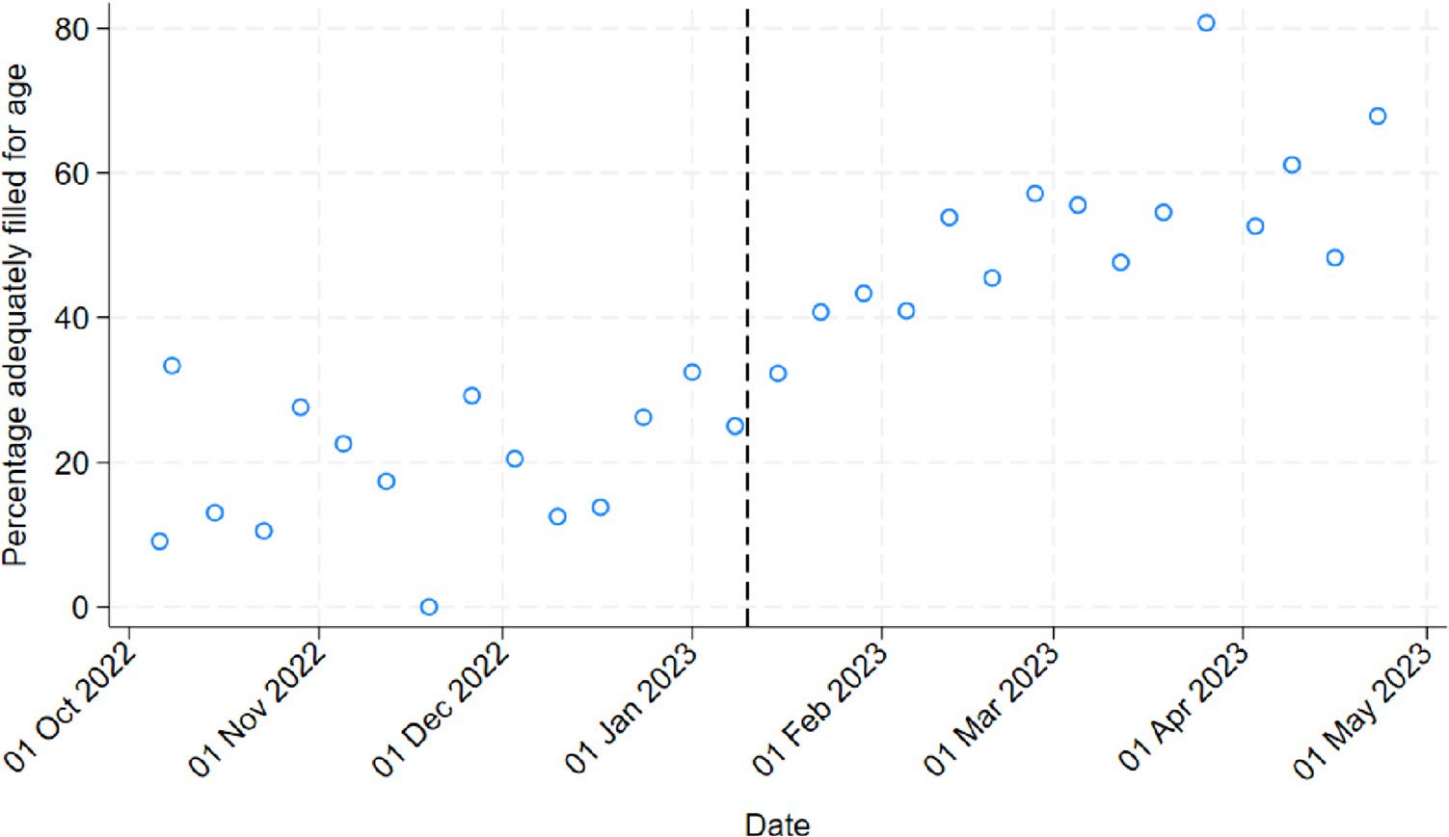
Percentage of blood culture samples adequately filled for age over time, dotted line denoting intervention start-point

Each age category saw a statistically significant improvement in adequate filling between phases, as shown in Table 2 and Fig. 2; however, the actual proportion adequately filled varied significantly between age categories (95.8% (69/72) of < 1 month, 73.4% (94/128) of 1 month–3 years and 22.9% (43/188) of > 3 years group). The proportion of adequately filled BCs over time is shown in Fig. 3.

In the regression analysis, adjusting for other factors, there was an 89% increase in volumes following introduction of the intervention (adjusted-incident-rate ratio 1.89 (95% CI 1.71–2.10), Online Supplemental Table 4), and a six-time higher odds of age-appropriate filling (adjusted odds ratio 6.20 (4.30–8.94), Online Supplemental Table 5). Sensitivity analysis, using an interrupted time-series accounting for trend-over-time, gave consistent results, indicating a significant increase of 0.80 ml in the blood volume post-intervention (Online Supplemental Tables 6 and Online Supplemental Fig. 1).

### Correlation of volume with other measures

Volume gradient bars were visible on 271 bottles (34.4% of total). Of these, 66.1% (179/271) had 9 gradient bars filled with actual blood volumes amongst those bottles ranging from 0.08 to 5.24 ml (Online Supplemental Fig. 2).

Estimated blood volume was provided in 144 post-intervention samples, and correlated well with actual volume, as seen in Online Supplemental Fig. 3 (PCC 0.77, *p* < 0.001). Only 38.9% (56/144) of estimates were within 0.2 ml of actual volume, with 38.9% (56/144) over-estimated, and 22.2% (32/144) under-estimated.

## Discussion

At study outset, BCs in our institution were under-filled, with nearly 30% under the minimum validated blood volume. This risks under-detection of pathogens and may cause false reassurance that a BC has been taken, when the likelihood of a positive result is minimal. Introducing an age-based policy, education and regular feedback has resulted in significant improvement, with an adjusted 89% increase in median volume and six-time higher odds of age-appropriate filling, adjusting for other factors, versus pre-intervention. Notwithstanding the sustained improvements seen post-intervention, there remains a significant proportion of BCs which are sub-optimally filled, particularly amongst older children, even using conservative age-specific guidelines.

Online Supplemental Table 7 summarizes interventional studies which have described pediatric BC volumes. Direct comparisons are limited by heterogeneous standards for adequacy and outcomes reported. Two interventional studies used the same age-appropriate guidelines as our study [26, 31]. Both reported higher rates of pre-intervention adequate filling than we described (46% and 53.9%, compared to 20.3%). One showed significant improvement post-intervention, but the other did not, and showed similar outcomes post-intervention to our study despite our lower baseline. There are some differences in our study populations, with both other studies being carried out in a tertiary children’s hospital, with over three times more cultures from central lines and all three studies finding line cultures attaining higher volumes.

Amongst pediatric interventional studies, our study shows the greatest increase in median blood volume (88% vs. 21–46%) [26, 32], which may be due to the active nature of our intervention with ongoing feedback and education, and lower baseline volumes. Interestingly, most studies have also found the lowest rates of adequate filling in older children [26, 31]. In our study, if the ≥ 3-year category is excluded, the proportion adequately filled for age rises from 47.3 to 91.6%. It is possible this is a function of simply having higher recommended volumes, and the difficulties of attaining higher volumes by collecting blood from cannula hub as is current practice, rather than a closed-venipuncture system such as that used in adults. Following discussions with sample takers, we plan to introduce closed-system venipuncture, recommend the use of 5 ml syringes as standard in this category, and monitor the effect of these changes.

At the other end of the age spectrum, two interventional studies have shown that it is possible to make significant improvements in the NNU median volume submitted, with our post-intervention median of 0.95 ml in line with other reports [33, 34]. In combination with a feasibility study [35], these studies show it is generally possible to obtain 1 ml even in the smallest infants, and we plan to emphasize this higher threshold in our guidance update.

During the study, blood volumes were a useful quality indicator used by clinical microbiologists and pediatricians in patient management; in a similar way, time-to-positivity and number of positive bottles have been used [36–38]. If there was a high suspicion for BSI, it was useful to know whether the admission BC was well-filled, while low blood volumes may increase the suspicion for sample contamination. Knowledge of BC volumes were also useful in follow-up cultures for *Candida* and *Staphylococcus aureus*, where negative BCs with low volumes were discounted when evaluating for evidence of pathogen clearance on treatment.

Given this practical usefulness, and evidence from other studies where volumes have declined in the years after monitoring ceases [26, 31], ongoing volume measurement is desirable. As our results indicate, neither BC gradient bars nor sample taker estimations are sufficiently accurate. Additionally, given a variance of 2.76 ml in BCs prior to blood sampling, exceeding volumes of many pediatric samples, pre-weighing of each individual bottle and matching that to post-inoculation weight is needed. Technological solutions may be imminent, with automated blood volume tracking processes included in newer BC systems [39]. Validation has been performed in adults, but further calibration of this system and validation for pediatric bottles are required [40, 41]. In older children, where there are higher required volumes, there may be a higher tolerance for variation between estimated and actual volumes, and these technologies may be more immediately implemented.

Our finding of a significantly higher proportion of adequately filled cultures detecting a pathogen, as assessed by under-filled BCs (9.4 vs. 2.9%), is in line with many others who have demonstrated a relationship between increasing blood volume and increasing pathogen detection [23, 26, 35] Several studies have also shown a relationship between lower blood volumes and increased contamination [31, 33]. In our study, over 80% of contaminants were from inadequately filled cultures. Potentially reducing contamination rates by increasing sample volumes would be beneficial as contaminants result in repeat sampling, unnecessary treatment, and prolonged length-of-stay, with associated healthcare costs and anxiety for families [42, 43].

As seen in this study even where there is particular focus on sample volumes, obtaining these may be challenging, particularly for older children. Therefore, new technologies may play a part in improving pathogen detection. The use of host-genomic biomarkers as diagnostic tools for serious bacterial infection shows early promise [44]. While these tests require significant further validation and will not yet provide the breadth of information as a bacterial isolate, they may prove a useful adjunct, especially where optimal volumes cannot be attained.

Another factor to consider is the number and type of BC bottles taken. As seen in Online Supplemental Digital Content 1, there are similar variations in these recommendations as for volume. Our institution uses a single pediatric bottle. Some recommend the additional routine use of anaerobic bottles [16, 23, 35]. However, anaerobic BSI in children is uncommon [45], and dividing sample into two bottles will reduce the volumes in each bottle, and may not increase diagnostic yield [14, 19, 35, 46]. As volumes increase, we plan to recommend anaerobic bottles for at-risk children only, as have others [18, 26, 31, 45].

There are several limitations to this study. Potential volume variations may have arisen as a result of variations of time between BC weighing and filling, as some have described evaporation reducing pre-inoculation volumes [26, 31] and the duration of incubation between receipt and weighing post-filling. Limiting stock releases to the ward, minimizing time from weighing to filling, and serial weighing of incubated BCs over 24 h without detecting volume variation minimized these potential variations. Given our focus on improving low sample volumes, we did not focus on the 5 ml upper limits of our BC bottle. In the post-intervention phase, 3.3% of samples were “overfilled,” which may impair BC performance, but was felt to be an acceptable risk in the context of low pre-intervention volumes.

## Conclusion

Optimizing BC inoculum volumes is key for the accurate diagnosis of BSI in children. Initial practice in our institution was sub-optimal, with only a third of BCs containing over 1 ml of blood. A multi-faceted intervention significantly improved volumes and demonstrated that with higher blood volumes, a higher proportion of BCs detect pathogens. Integrating feedback into routine reporting of BCs, possibly through automated systems, may allow for the use of BC monitoring as a quality indicator in practice, to improve diagnostic performance.

### Supplementary Information

Below is the link to the electronic supplementary material.

Supplementary file1 (DOCX 4100 KB)

## Data Availability

No datasets were generated or analyzed during the current study.

## Abbreviations

BC: Blood cultures
BSI: Bloodstream infections
ED: Emergency department
IQR: Inter-quartile range
PCC: Pearson correlation coefficient
NNU: Neonatal unit
SHO: Senior House Officer
