# Supplementary material for: Pediatric blood cultures—turning up the volume: a before and after intervention study
Source: Eur J Pediatr. 2024 Apr 24;183(7):3063–71. doi: 10.1007/s00431-024-05544-0 (PMC11192679; doi:10.1007/s00431-024-05544-0)
Supplement: Supplementary file 1 — Supplementary file1 (DOCX 4100 KB) [file 431_2024_5544_MOESM1_ESM.docx]

Paediatric blood cultures – turning up the volume: a before and after intervention study

Seán Olann Whelan MB BAO BCh^1,2^*, Conor Mulrooney MB BAO BCh^1^, Frank Moriarty PhD^3^, Martin Cormican MD, FRCPath^1,4^

^1^ Division of Medical Microbiology, Galway University Hospital, Galway, Ireland

^2^ Department of Microbiology, Children’s Health Ireland at Temple Street, Dublin, Ireland

^3^ School of Pharmacy and Biomolecular Sciences, RCSI University of Medicine and Health Sciences, Dublin, Ireland

^4^ Discipline of Bacteriology, College of Medicine, Nursing & Health Sciences, University of Galway, Galway

*Corresponding author [s.whelan@rocketmail.com](mailto:s.whelan@rocketmail.com),

**Online Supplemental Table 1.** Summary of (a) weight-based, (b) age-based and (c) universal recommendations for paediatric blood culture volumes, updated from Huber et al ^39^.

| 1. ***Weight-based*** | **Weight (kg)** | **Total blood volume (ml)** | **No of bottles** |
| --- | --- | --- | --- |
| Kellogg et al. ^16,17^ | ≤1.0 | 2.0 | 2 |
|  | 1.1 - 2.0 | 4.5 | 2 |
|  | 2.1 - 12.7 | 6.0 | 2 |
|  | 12.8 - 36.3 | 23.0 | 4 |
|  | >36.3 | 60.0 | 4 |
|  |  |  |  |
| Gaur et al. ^22^ | 1.5 - 2.1 | 1.0 | 1 |
|  | 2.2 - 11.1 | 1.5 | 1 |
|  | 11.2 - 17.1 | 7.5 | 3 |
|  | 17.2 - 37.2 | 11.5 | 3 |
|  | ≥37.3 | 16.5 | 3 |
|  |  |  |  |
| Gonsalves et al. ^23^ | ≤3.9 | 1.0 | 2 |
|  | 4.0 - 7.9 | 3.0 | 2 |
|  | 8.0 - 13.9 | 6.0 | 2 |
|  | 14.0 - 18.9 | 12.0 | 4 |
|  | 19.0 - 25.9 | 16.0 | 4 |
|  | 26.0 - 39.9 | 20.0 | 4 |
|  | 40.0 - 53.9 | 32.0 | 4 |
|  | ≥54 | 40.0 | 4 |
|  |  |  |  |
| Revell & Doern ^8^ | ≤3 | 2.0 | 1 |
|  | >3.0 - 5.0 | 3.0 | 1 |
|  | >5.0 - 7.0 | 5.0 | 1 |
|  | >7.0 - 12.0 | 10.0 | 2 |
|  | >12.0 - 20.0 | 15.0 | 3 |
|  | >20.0 - 30.0 | 30.0 | 3 |
|  | >30.0 - 45.0 | 40.0 | 4 |
|  | >45.0 | 60.0 | 6 |
|  |  |  |  |
|  |  |  |  |
| El Feghaly et al. ^24^ | ≤5.0 | 1.0 | 1 |
|  | 5.1 - 10.0 | 2.0 | 1 |
|  | 10.1 - 20.0 | 6.0 | 2 |
|  | 20.1 - 40.0 | 10.0 | 2 |
|  | >40.0 | 20.0 | 2 |
|  |  |  |  |
| Miller et al. ^27^ | ≤1.0 | 2.0 | Not specified |
|  | 1.1-2.0 | 4.0 | Not specified |
|  | 2.1-12.7 | 6.0 | Not specified |
|  | 12.8-36.3 | 20.0 | Not specified |
|  | >36.3 | 40 - 60 | Not specified |
|  |  |  |  |
| Kaditis et al. ^28^ | <1.5 | 1.0 | 1 |
|  | 1.5 - 3.9 | 2.0 | 2 |
|  | 4.0 - 7.0 | 6.0 | 4 |
|  | 8.0 - 13.0 | 9.0 | 6 |
|  | 14.0 - 18.0 | 23.0 | 6 |
|  | 19.0 - 25.0 | 30.0 | 6 |
|  | 26.0 - 39.0 | 40.0 | 6 |
|  | ≥40.0 | 60.0 | 6 |
|  |  |  |  |
| 1. ***Age-based*** | **Age** | **Total blood volume [ml]** | **No of bottles** |
| Sarkar et al. ^25^ | <1 month | ≥1.0 | 1 |
|  |  |  |  |
| Yaacobi et al. ^14^ | <1 month | 1.0 | 2 |
|  |  |  |  |
| Connell et al. & Harewood et al. ^26,31^ | <1 month | >0.5 | 1 |
|  | ≥1 - 36 months | ≥1.0 | 1 |
|  | >36 months | ≥4.0 | 1 - 2 |
|  |  |  |  |
| Revell & Doern ^8^ | <12 months | 1.0 - 3.0 | 1 - 2 |
|  | 12 - 48 months | 3.0 - 4.0 | 1 - 2 |
|  | 5 - 9 years | 6.0 - 8.0 | 1 - 2 |
|  | ≥10 years | 20.0 | 2 |
|  |  |  |  |
| 1. ***Universal*** |  | **Total blood volume [ml]** | **No of bottles** |
| BD Bactec Specimen Collection Guidance ^13^ | Range | 0.5 - 5.0 | 1 |
|  | Optimal | 1.0 - 3.0 | 1 |

**Online Supplemental Image 1.** Poster in all clinical areas summarising newly introduced guidance as part of intervention.


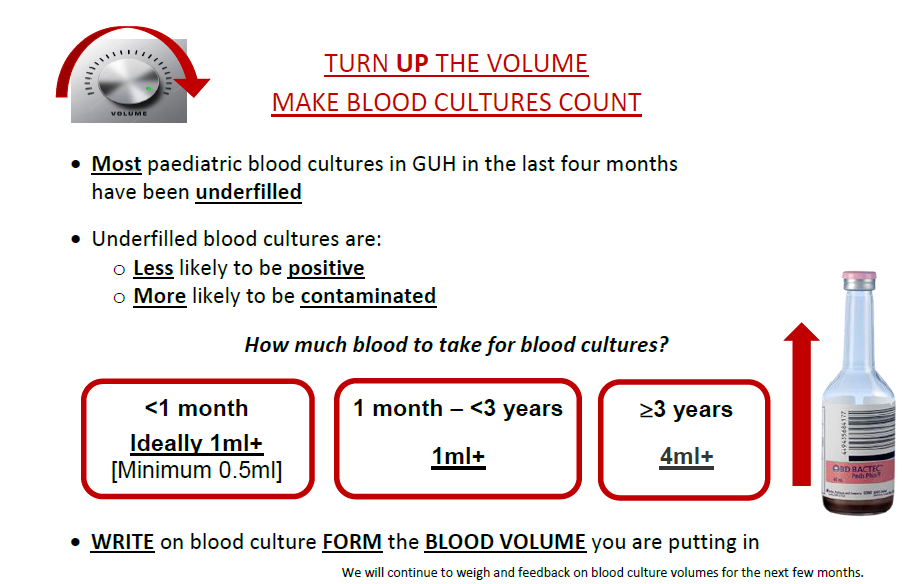


**Online Supplemental Table 2.** Blood volumes per patient characteristic.

| Characteristics | Blood volume (ml), median (IQR) | p value* |
| --- | --- | --- |
| **Age category** |  | <0.001 |
| <1month | 0.72 (0.46-1.10) |  |
| 1month-3years | 0.91 (0.50-1.52) |  |
| >3 years | 1.69 (0.98-3.07) |  |
| **Location** |  | <0.001 |
| ED | 1.10 (0.59-1.98) |  |
| NNU | 0.66 (0.44-1.05) |  |
| Ward | 1.56 (1.04-2.64) |  |
| **Gestational age category** |  | 0.83 |
| Very preterm (<32 weeks) | 0.59 (0.23-1.23) |  |
| Moderate-late preterm (32-37 weeks) | 0.65 (0.46-0.98) |  |
| Term (>37 weeks) | 0.79 (0.44-1.08) |  |
| **Sampler** |  | <0.001 |
| Nurse | 1.87 (1.05-2.84) |  |
| Registrar | 0.90 (0.52-1.51) |  |
| SHO | 1.31 (0.75-2.44) |  |
| **Site** |  | <0.001 |
| Peripheral | 1.05 (0.57-1.80) |  |
| Central | 2.10 (1.33-2.85) |  |
| **BC Results:** |  |  |
| **Positive** | 1.14 (0.60-1.96) |  |
| **Significance:** |  | <0.001 |
| Contaminant | 0.54 (0.35-1.12) |  |
| Pathogen | 1.85 (1.06-3.23) |  |

* p value for difference in median blood-volume across categories based on the Kruskal-Wallis test.

**Online Supplementary Table 3.** Organisms identified from positive blood cultures, divided by significance. Note: Overall and individual numbers will not match directly, as some cultures were poly-microbial.

| **Significant [n=38]** | **n** |
| --- | --- |
| *Staphylococcus epidermidis* | 8 |
| *Staphylococcus aureus* | 7 |
| *Candida albicans* | 6 |
| *Pseudomonas aeruginosa* | 6 |
| *Staphylococcus capitis* | 3 |
| *Acinetobacter lwoffii* | 2 |
| *Klebsiella pneumoniae* | 2 |
| *Streptococcus pyogenes* | 2 |
| *Enterobacter cloacae* | 1 |
| *Streptococcus mitis* | 1 |
| **Contaminants [n=25]** | **n** |
| Coagulase negative staphylococci | 14 |
| Gram positive bacilli - unidentified | 2 |
| *Corynebacterium striatum* | 1 |
| *Micrococcus luteus* | 2 |
| *Moraxella catharrhalis* | 1 |
| *Pantoea agglomerans* | 1 |
| *Rosemonas mucosa* | 1 |
| *Viridans group streptococcus* | 2 |
| *Acinetobacter* species | 1 |
| *Brevibacterium* species | 1 |

| **Online Supplemental Table 4.** Regression analysis for change in rate of blood volume. | | | |
| --- | --- | --- | --- |
|  | Coefficient | 95% CI | p-value |
| **Intervention** | 1.89 | [1.71, 2.10] | <0.01 |
| **Age (years)** | 1.07 | [1.05, 1.08] | <0.01 |
| **Staff grade (Reference – Registrar)** | 1.00 |  |  |
| Nurse | 1.20 | [0.98, 1.47] | 0.07 |
| SHO | 1.14 | [1.02, 1.27] | 0.02 |
| **Clinical area (Reference – ED)** | 1.00 |  |  |
| NNU | 0.73 | [0.64, 0.83] | <0.01 |
| Ward | 0.95 | [0.84, 1.08] | 0.44 |
| **Central line (Reference – Peripheral)** | 1.17 | [0.95, 1.46] | 0.14 |

| **Online Supplemental Table 5.** Regression analysis for odds of sample being adequately filled for age. | | | |
| --- | --- | --- | --- |
|  | Coefficient | 95% CI | p-value |
| **Intervention** | 6.20 | [4.30, 8.94] | <0.01 |
| **Age (years)** | 0.83 | [0.79, 0.88] | <0.01 |
| **Staff grade (Reference – Registrar)** | 1.00 |  |  |
| Nurse | 0.56 | [0.27, 1.14] | 0.11 |
| SHO | 1.10 | [0.75, 1.62] | 0.63 |
| **Clinical area (Reference – ED)** | 1.00 |  |  |
| NNU | 3.19 | [1.85, 5.51] | <0.01 |
| Ward | 0.83 | [0.53, 1.33] | 0.44 |
| **Central line (Reference – Peripheral)** | 2.38 | [1.19, 4.74] | 0.01 |

| **Online Supplemental Table 6.** Interrupted times series regression for blood volume accounting for trend. | | | |
| --- | --- | --- | --- |
|  | Coefficient | 95% CI | p-value |
| Trend pre-intervention | -0.000 | [-0.008, 0.008] | 0.94 |
| Immediate change post-intervention | 0.799 | [0.162, 1.436] | 0.02 |
| Trend change post-intervention | 0.003 | [-0.008, 0.013] | 0.79 |

**Online** **Supplemental Figure 1** Interrupted time series accounting for trend-over-time. Dotted line denoting intervention start-point.


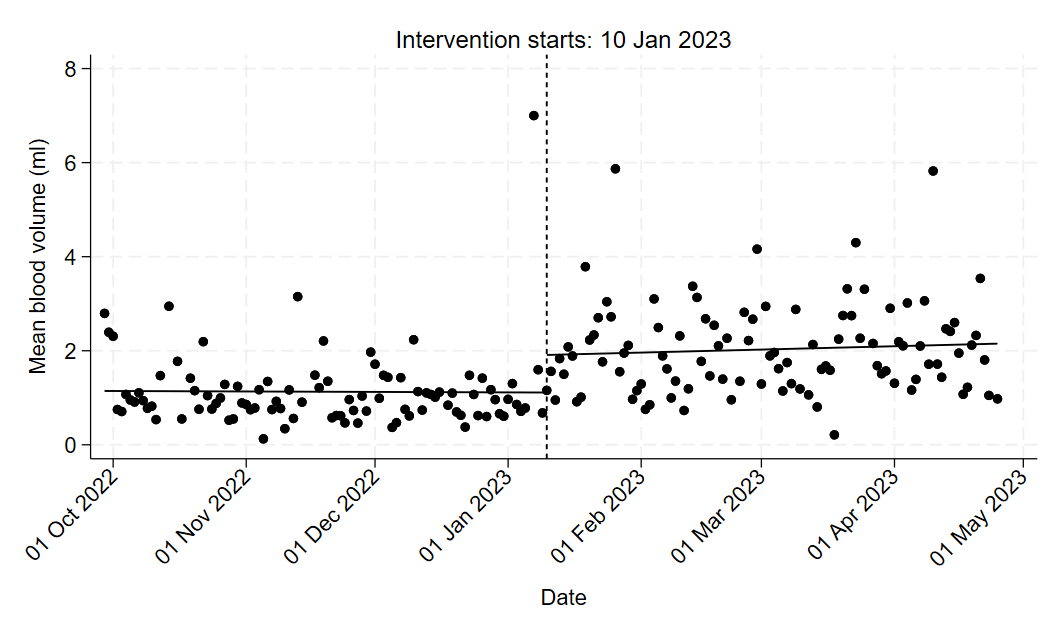


**Online Supplemental Figure 2.** Violin plot showing blood culture blood-volumes (ml) at each bottle gradient-bar.


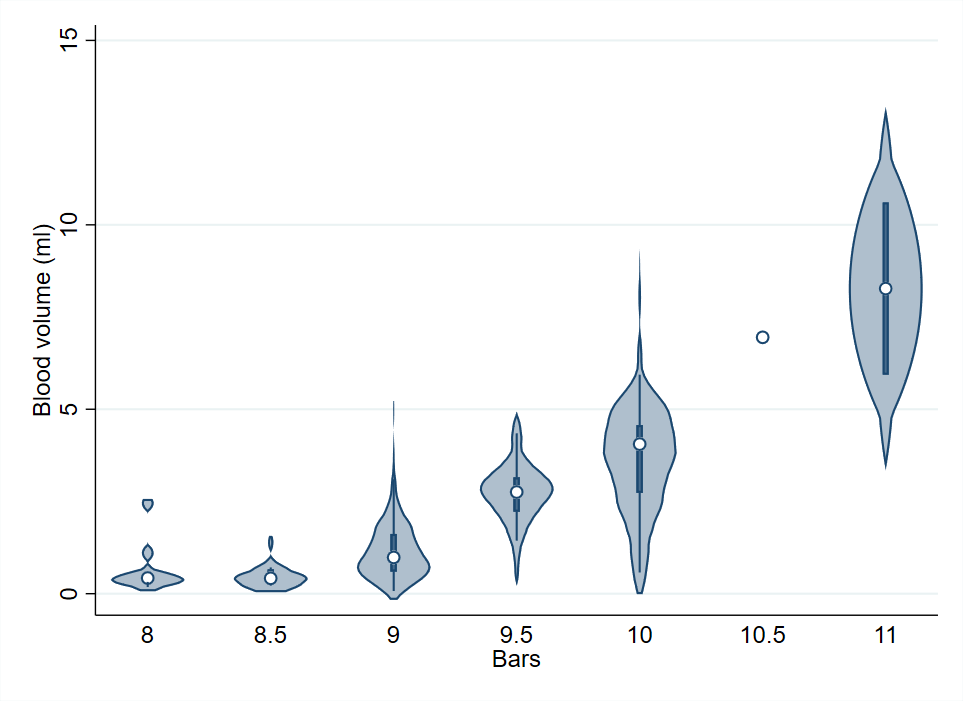


**Online Supplemental Figure 3.** Scatterplot illustrating correlation between estimated and actual BC blood-volumes.


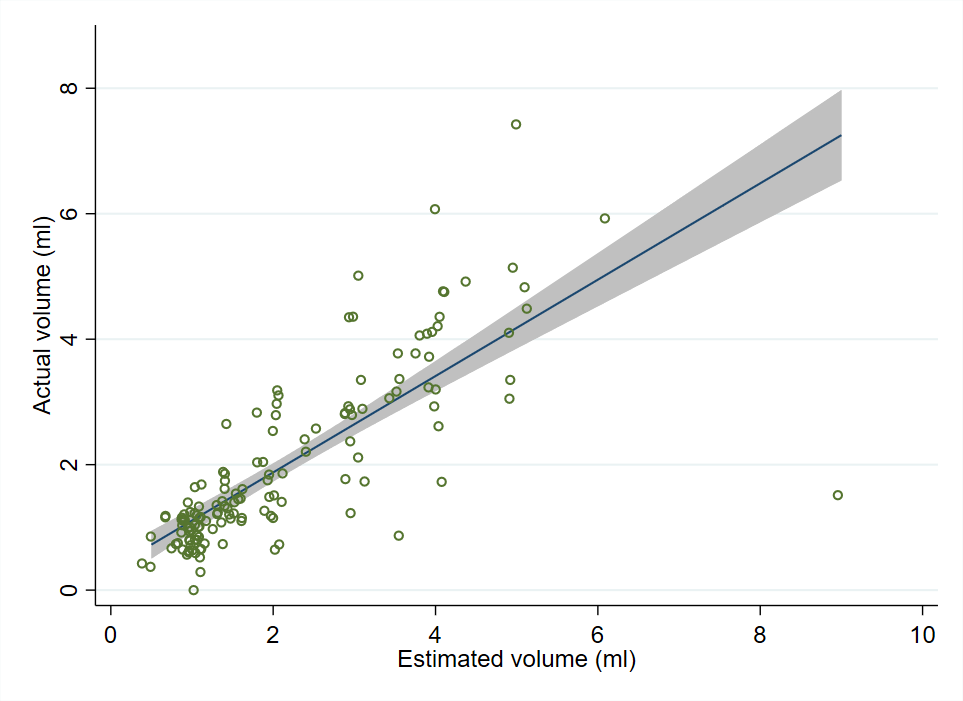


**Online Supplemental Table 7.** Overview of other interventional studies targeting paediatric blood-culture blood volumes.

| Study | Population | Standard for adequately filled | | Pre-intervention | Post-intervention | Details of intervention | Culture results |
| --- | --- | --- | --- | --- | --- | --- | --- |
| **Connell et al.** ^26^ | Tertiary children’s hospital | Age | Volume blood (mL) | 1067 cultures | 291 cultures | Introduced posters | 7.4% positive, 50% contaminants |
|  |  | <1 month | >0.5 | 46% adequate volume | 63.9% adequate volume* |  | 5.2% Adequate volume positive,  2.2% inadequate volume positive* |
|  |  | ≥1-36 months | ≥1.0 |  |  |  |  |
|  |  | >36 months | ≥4.0 |  |  |  |  |
|  |  |  |  |  |  |  |  |
| **Harewood et al.** ^31^ | Tertiary children’s hospital | As above | | 2939 cultures | 2188 cultures | Targeted education | 6% positive, 22% contaminants |
|  |  |  |  | 53.9% adequate volume | 51.4% adequate volume | Lanyard-card introduction | True-positive = 4.4% adequate volume  vs 5% inadequate |
|  |  |  |  |  |  |  | Contaminant = 0.8% adequate volume  vs 1.9% inadequate* |
|  |  |  |  |  |  |  |  |
| **Allen et al.** ^34^ | NNU | ≤1000g | ≥0.5ml | No detail on number of cultures/adequacy | 450 cultures | Guideline introduced | Contamination rates decreased  from 2% to 1%* |
|  |  | >1000g | ≥1ml | Range of 0.3-1.5mL | Adequate 94%, mean 1.1ml | Neonatal BC sticker |  |
|  |  |  |  |  |  | BC record-book creation |  |
|  |  |  |  |  |  |  |  |
| **Ohnishi et al.** ^32^ | Paediatrics - general hospital | All except neonates | 3ml | 331 cultures | 996 cultures | Guideline introduced | True positive = 2.7% pre-intervention,  1.95% post-intervention |
|  |  |  |  | Median 1.73ml [IQR 1.20ml-2.52ml) | Median 2.54ml (1.69ml-3.39ml)* | Periodic staff reminders | Contaminants = 0.6% preintervention,  0.9% post-intervention |
|  |  |  |  |  |  |  | No difference positive rate  between >1ml and <1ml |
|  |  |  |  |  |  |  | True-positive median 2.33ml,  Contaminant 2.81ml |
|  |  |  |  |  |  |  |  |
| **Singh et al.** ^33^ | NNU | Target 1ml, accepting ≥0.8ml | | 130 cultures | 507 cultures | Guideline introduced | 5% true-positive,  1.6% contaminants |
|  |  |  |  | 96.9% suboptimal | 25.1% suboptimal* | Label for notes | True-positive = 5.5% optimal volume vs 4.4% suboptimal |
|  |  |  |  | Median volume = 0.36ml | Median volume = 0.9ml* | Education sessions | Contaminants = 0.5% optimal volume vs 3.2% suboptimal * |

*Denotes significant change
